# Supplementary material for: The interplay between cognitive biases, attention control, and social anxiety symptoms: A network and cluster approach
Source: PLoS One. 2023 Apr 7;18(4):e0282259. doi: 10.1371/journal.pone.0282259 (PMC10081794; doi:10.1371/journal.pone.0282259)
Supplement: S1 File — (DOCX) [file pone.0282259.s001.docx]

**Supporting Information**

**Measures: Additional Information**

**SST**

New sentences adhere to restrictions of the original SST, i.e., half of the emotional sentences show the positive before the negative word, and target words never follow directly after one another or start/end a scrambled sentence. Additionally, this version of the SST takes into account that German grammar often allows for more than one correct word order.

**VST**

Stimuli were taken from the FACES database (Ebner et al., 2010). This set of faces is well validated and highly reliable. Two female and two male models were selected, with a young adult and a middle-aged model of each gender. To standardize these four models, pictures were cropped to remove non-facial areas and produce oval shapes with identical measures (Calvo et al., 2008; Williams et al., 2005). Brightness and contrast were adjusted and faces were presented in grayscale on a white background (each 20% of screen in height).

**ANT**

First, participants see a fixation cross at the center of the display (remaining 400-1600ms). This fixation cross is presented for another 100ms (no-cue condition) or is replaced by an asterisk cue (central-cue condition) at 5% of screen in height. There are two more conditions for cue allocations; double-cue condition shows an asterisk cue above *and* below the fixation cross, and spatial-cue condition shows a single asterisk cue above *or* below the fixation. After another fixation interval of 400ms, targets are shown until a response is given or for up to 1700ms. Five horizontally arranged stimuli (a central target arrow surrounded by two flanking arrows on each side) are presented directly above or below the fixation cross, with each arrow at 6% of screen in height. Flanking arrows point either in the same (congruent flanking) or the other direction (incongruent flanking) as the target arrow or no direction at all (neutral flanking). Participants must indicate the direction of the target arrow by giving a left-or-right response. In total, this design includes 4 x 3 x 2 x 2 conditions which are displayed in random order: 4 cue conditions (no cue, center cue, double cue, spatial cue), 3 flanking conditions (neutral, congruent, incongruent), 2 target positions (above or below fixation cross), 2 target directions (left or right).

**Figures**

**
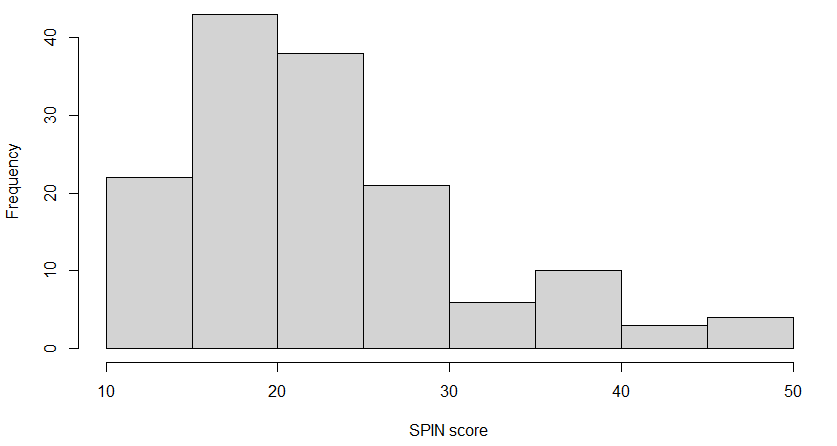
**

**S1 Fig. Histogram of SPIN score distribution in the current sample.** SPIN = Social Phobia Inventory.

| 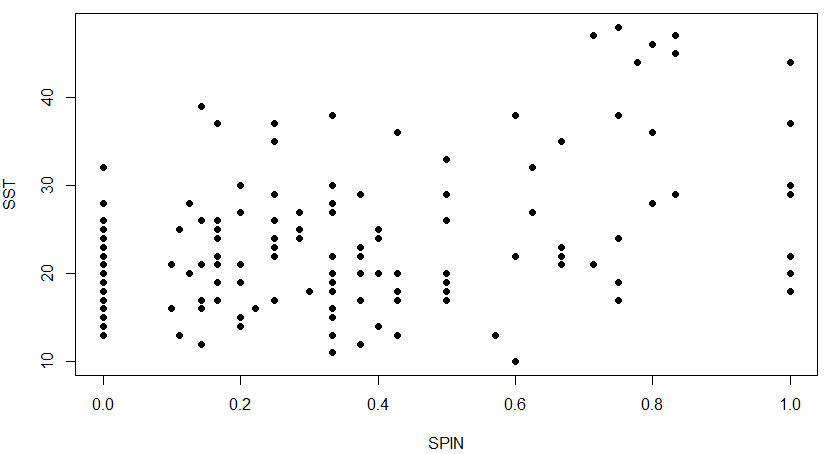 | 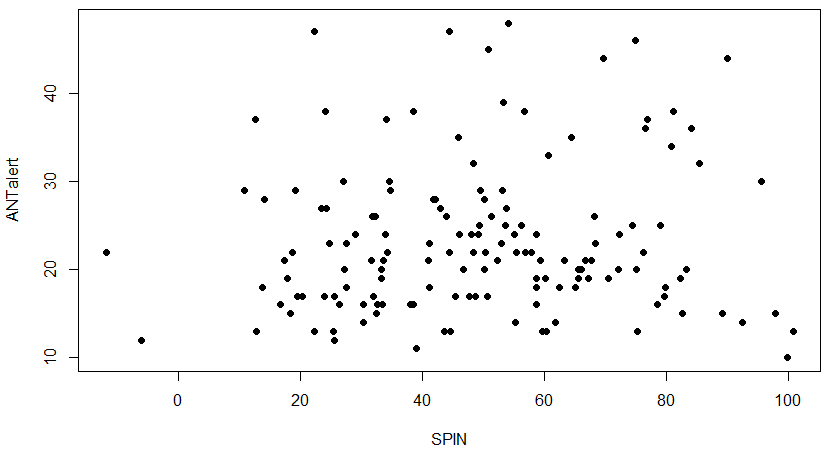 |
| --- | --- |
| 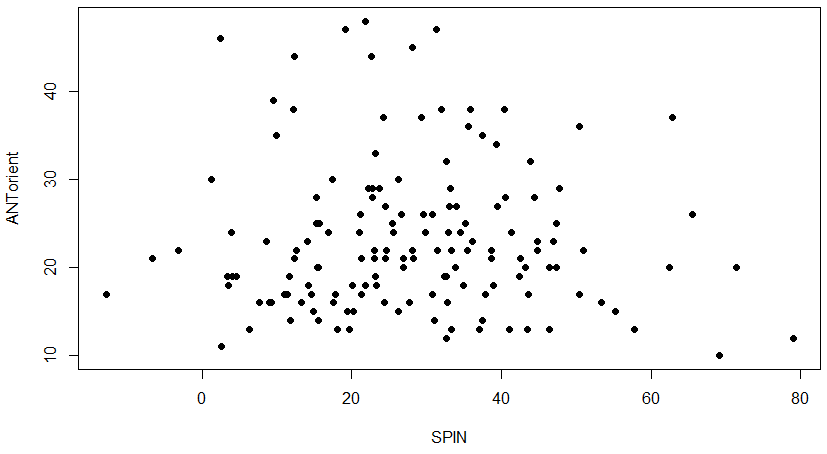 | 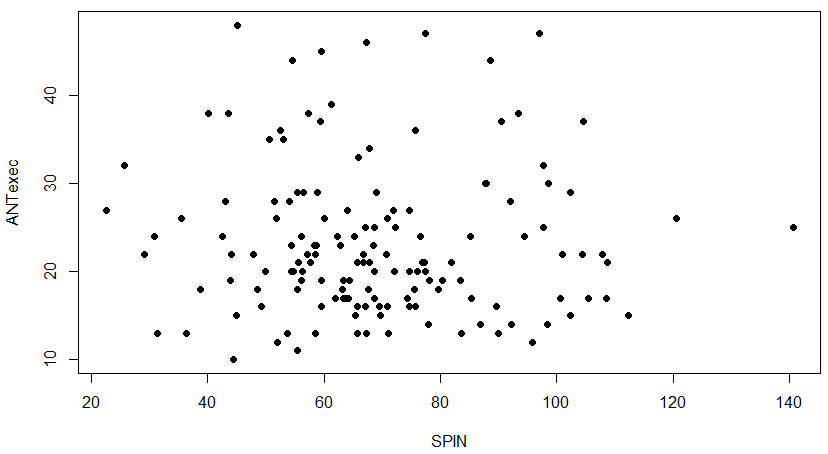 |
| 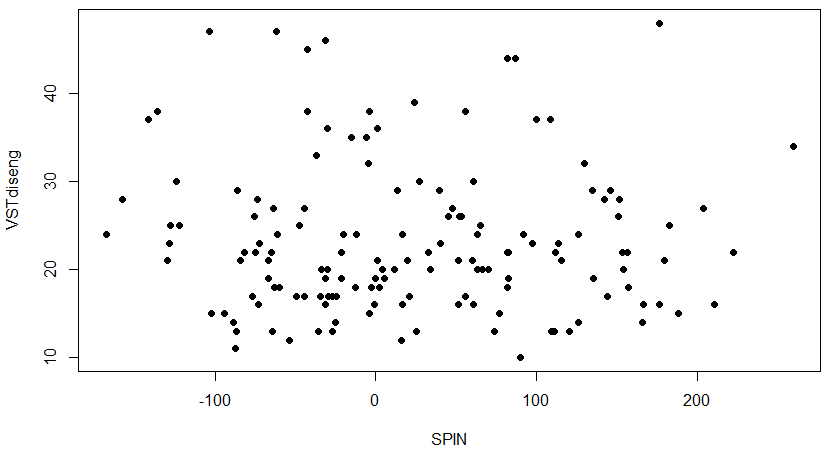 | 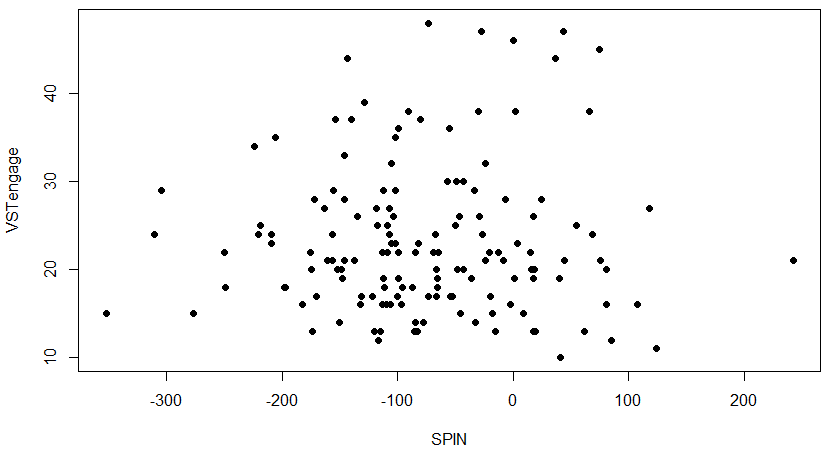 |

**S2 Fig. Scatter plots of SPIN score distribution and respective cognitive (dys)functions.** ANT: Alert, Orient, Execut = Attention Network Test: Alerting, Orienting, Executive function. VST: Diseng, Engage = Visual Search Task: disengagement, engagement; SST = Scrambled Sentence Task; SPIN = Social Phobia Inventory.


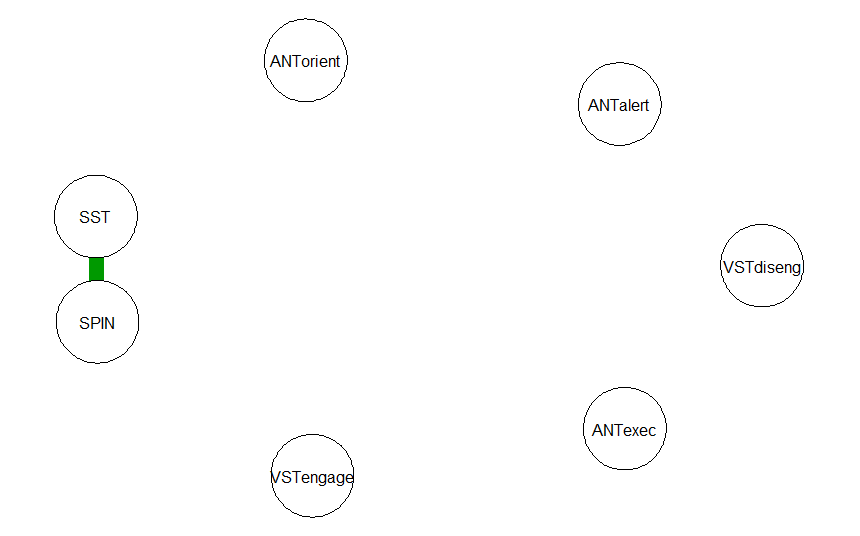


**S3 Fig. Diagram of Partial Correlation Network with Bonferroni Correction.** ANT: Alert, Orient, Execut = Attention Network Test: Alerting, Orienting, Executive function. VST: Diseng, Engage = Visual Search Task: disengagement, engagement; SST = Scrambled Sentence Task; SPIN = Social Phobia Inventory.


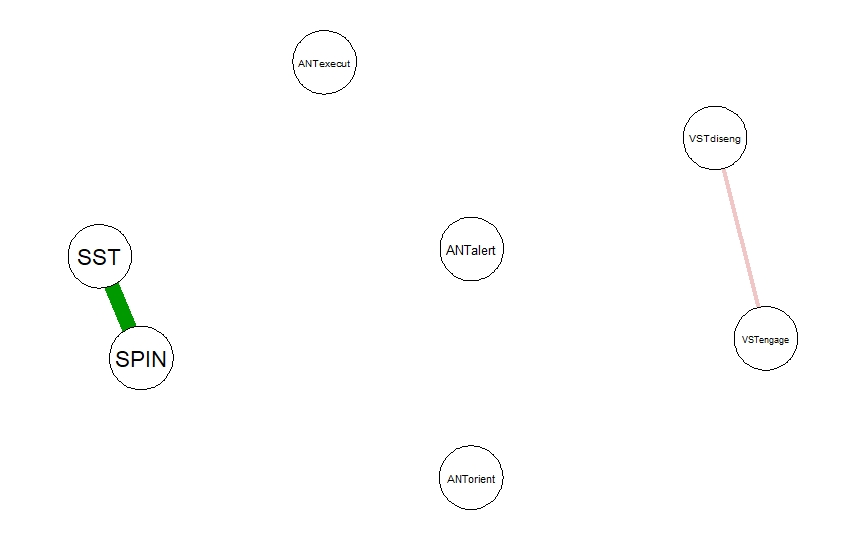


**S4 Fig. Diagram of Partial Correlation Network with Tuning Parameter Set to ɣ = 0.** ANT: Alert, Orient, Execut = Attention Network Test: Alerting, Orienting, Executive function. VST: Diseng, Engage = Visual search task: disengagement, engagement; SST = Scrambled Sentence Task; SPIN = Social Phobia Inventory.


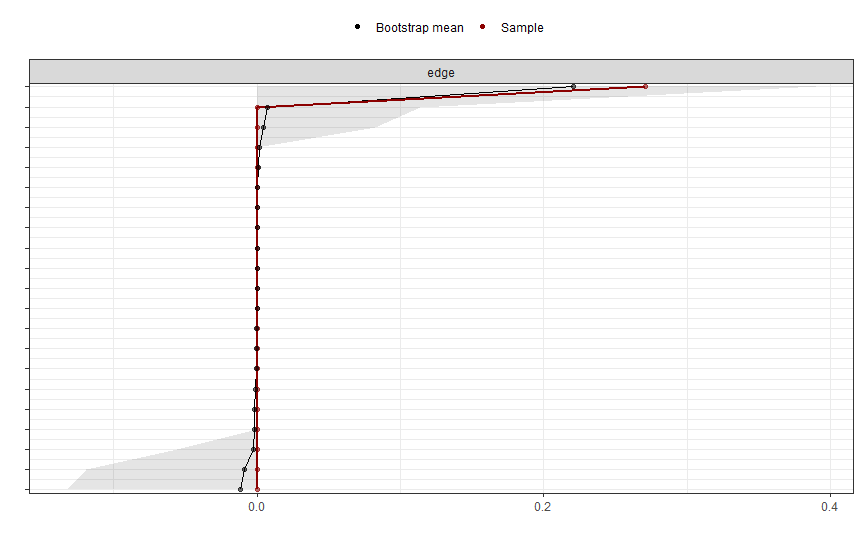


**S5 Fig.** **Bootstrapped confidence intervals of estimated edge-weights.** Non-parametric bootstrap (2500 samples). Black is bootstrap mean, red is sample.


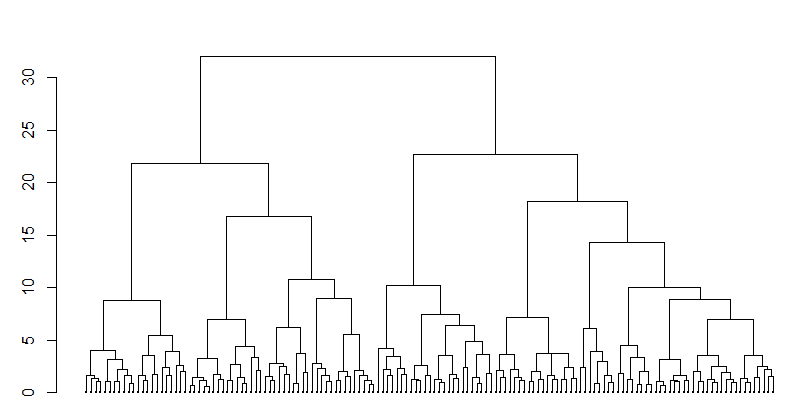


**S6 Fig. Dendrogram from Hierarchical Clustering.**

**Tables**

**S1 Table. Correlation Matrix Used for Power Analysis.**

| Variable | VST | SST | ANT Alert | ANT Orient | ANT Exec | SPIN |
| --- | --- | --- | --- | --- | --- | --- |
| VST | 1 |  |  |  |  |  |
| SST | .28^1^ | 1 |  |  |  |  |
| ANT Alert | -.20^2^ | .00^1^ | 1 |  |  |  |
| ANT Orient | -.20^2^ | .00^1^ | -.24^4^ | 1 |  |  |
| ANT Exec | .00^2^ | .00^1^ | -.02^4^ | -.15^4^ | 1 |  |
| SPIN | .24^5^ | .29^3^ | .09^4^ | -.34^4^ | -.02^4^ | 1 |

Correlations taken from: ^1^ Everaert, Duyck, & Koster, 2014; ^2^ Heeren & McNally, 2016; ^3^ Huppert, Foa, Furr, Filip, & Mathews, 2003; ^4^ Moriya & Tanno, 2009; ^5^ Van Bockstaele et al., 2011. VST (Visual Search Task), SST (Scrambled Sentence Task), ANT (Attentional Network Task) and SPIN (Social Phobia Inventory).

**S2 Table. Means and SDs of Cognitive Measures by Groups.**

|  | Group 1 |  | Group 2 |  | Group 3 |  | *F*(2,144) | Contrast  (Tukey HSD) |
| --- | --- | --- | --- | --- | --- | --- | --- | --- |
|  | M | SD | M | SD | M | SD |  |  |
| ANT: Alert | 0.34 | 0.91 | -0.28 | 0.94 | -0.18 | 1.14 | 7.0 | 1 = 2 > 3 |
| ANT: Ort | 0.01 | 1.02 | -0.41 | 0.72 | 0.97 | 0.86 | 21.8 | 1 > 3 = 2 |
| ANT: Exc | -0.24 | 0.90 | 0.38 | 1.11 | -0.32 | 0.66 | 8.1 | 1 = 2 = 3 |
| VST: Diseng | -0.47 | 0.69 | 0.02 | 0.96 | 1.13 | 0.84 | 33.16 | 3 > 1 = 2 |
| VST: Engag | 0.06 | 1.11 | 0.03 | 0.90 | -0.21 | 0.94 | 0.7 | 2 = 1 > 3 |
| SST | 0.72 | 0.97 | -0.54 | 0.51 | -0.48 | 0.86 | 44.16 | 2 > 1 = 3 |
| SPIN | 0.29 | 1.13 | -0.30 | 0.75 | -0.01 | 1.00 | 5.6 | 2 > 1 = 3 |

Multiple comparisons are controlled for alpha = 0.05.
