# Supplementary material for: The interplay between cognitive biases, attention control, and social anxiety symptoms: A network and cluster approach
Source: PLoS One. 2023 Apr 7;18(4):e0282259. doi: 10.1371/journal.pone.0282259 (PMC10081794; doi:10.1371/journal.pone.0282259)
Supplement: Attachment — Submitted filename: review letter.docx [file pone.0282259.s002.docx]

The manuscript "The Interplay Between Cognitive Biases, Attention Control, and Social Anxiety Symptoms: A Network and Cluster Approach" outlines a study designed to investigate the interrelationship between cognitive biases and executive dysfunctions in a network and cluster approach. This study addresses an important topic, in that the causes and maintenance mechanism of social anxiety is increasingly being examined and tested. In general, the study design is clear and is written in good english. However, I do have some concerns regarding the study analyses, and interpretation of findings.

1. The odd-even reliability for the engagement and disengagement score was too low, r = .12 and .08 respectively, which limits the stability and generalizability of the reported results.
2. The results reported in this study are clearly contrary to those of a recent study on a similar topic, and I hope the authors can give a reasonable explanation.

Leung, C. J., Yiend, J., & Lee, T. M. (2022). The Relationship Between Attention, Interpretation, and Memory Bias During Facial Perception in Social Anxiety. Behavior Therapy.

3. The distribution of anxiety scores of the subjects was not provided, such as in a scatter plot. What is the proportion of the number of high and low social anxiety people? Which may affect the explanation of the experimental results.
